# Distribution of Protein Content and Number of Aggregates in Monoclonal Antibody Formulation After Large-Scale Freezing

**DOI:** 10.1208/s12249-018-1281-z

**Published:** 2019-01-10

**Authors:** Astrid Hauptmann, Georg Hoelzl, Thomas Loerting

**Affiliations:** 1 Institute of Physical Chemistry, University Innsbruck, Innrain 52c, 6020 Innsbruck, Austria; 2 Sandoz GmbH, Biochemiestraße 10, 6336 Langkampfen, Austria

**Keywords:** aggregation, mAb, cryoconcentration, freeze-concentrated solution, large-scale freezing

## Abstract

Cryoconcentration of an in-house IgG_1_ and number of aggregates in a formulation containing trehalose were determined in dependence on freezing protocol and volume. Morphology changes of ice crystals depending on cooling rates were captured by optical cryomicroscopy (OCM) images. UV-Vis and affinity chromatography (ALC) was used to determine protein content and size-exclusion chromatography (SEC) for detection of aggregates. Cooling to − 80°C rather than − 20°C is beneficial in avoiding hot spots of high protein concentration. An upscaling of 250 ml to 2 L bottles results in an up to fourfold increase of macroscopic cryoconcentration. There is no direct correlation between number of aggregates and macroscopic cryoconcentration. Aggregate formation of that specific mAb is not caused by macroscopic cryoconcentration but can be directly linked to microscopic cryoconcentration in between the ice dendrites. Slower cooling with set-point and storage temperatures below T_g_’ has proven to be advantageous for the prevention of aggregate formation. We reveal that the subcooling prior to freezing plays a key role in avoiding aggregates. The lower the solution is supercooled the more likely aggregates form. As a consequence, we suggest controlled initiation of the freezing process to avoid large supercooling.

**Electronic supplementary material:**

The online version of this article (10.1208/s12249-018-1281-z) contains supplementary material, which is available to authorized users.

## INTRODUCTION

**Fig. 1:**
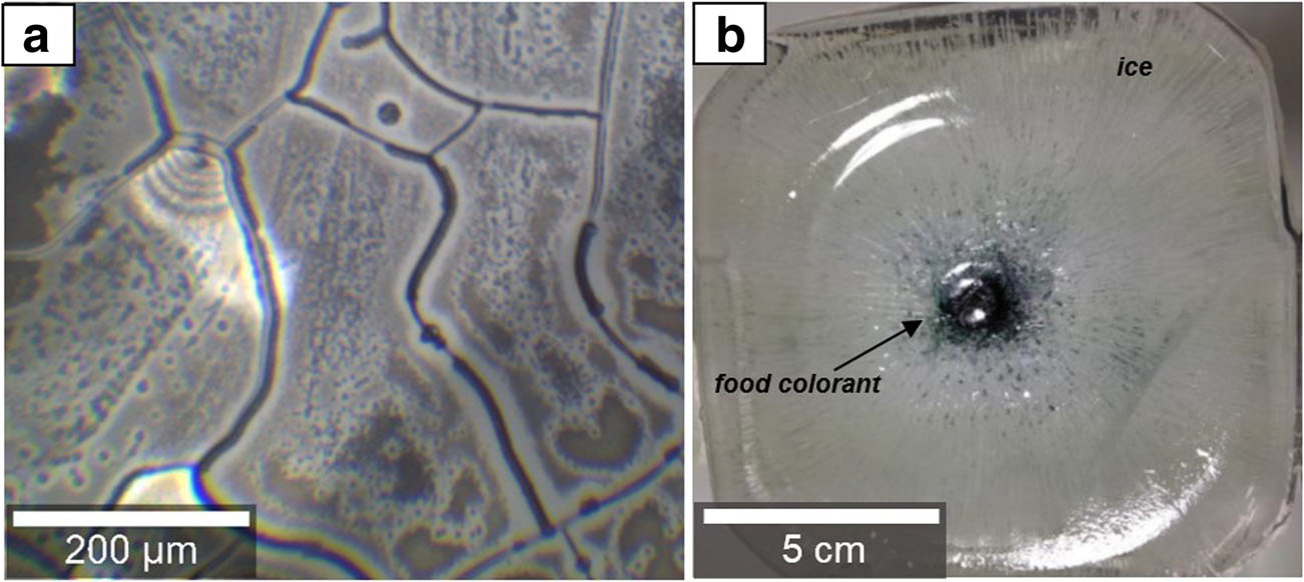
Exemplary cryomicroscopy images showing the effects of two types of cryoconcentration: **a** “Amorphous phase freeze-concentration” of mAb solution at − 80°C and **b** “bulk-scale/progressive freeze-concentration” of food colorant in water at − 80°C. Note the different length scales in the two images

From the first licensed monoclonal antibody (mAb) product in 1986 (1) until now, the therapy with mAb solutions has evolved. Today, over 100 different known monoclonal antibodies approved by the FDA are successfully implemented in the market (2). From the production of the drug substance to the storage and further transportation of the drug product pharmaceutical companies are constantly experiencing significant technical challenges that have to be considered. One of these challenges is finding a suitable freeze and thaw process (FT), optimizing time and cost efficiency while maintaining purity, activity, and efficacy of the final product. Pharmaceutical industries are currently using several methods to freeze therapeutic protein solutions which vary in volume capacities, geometry, container material, and handling. Cryovessels/cryowedge (Sartorius-Stedim Biotech, France) (3,4), freeze container (ZetaHolding, Austria), and Celsius bags (Sartorius-Stedim) (5) are actively temperature controlled by circulating a coolant fluid. Another option is to use bottles such as carboys which are passively temperature controlled by placing them into freezers. There are certain benefits regarding the freezing of drug substance that outweigh the disadvantages. Those benefits include the increased product stability and shelf life, decreased microbial growth due to low temperatures, and the prevention of foam formation during transportation. Also, chemical degradation reactions, such as deamidation, hydrolysis, or oxidation are slowed down (3). Disadvantages include possible stress for proteins through cold denaturation (6,7) or pH-shifts due to freeze-concentration (8,9), to name just two. It had also been shown that the ice-liquid interface may pose a problem for proteins (10–12), *e.g.*, some proteins are adsorbed on the surface of ice crystals and undergo unfolding/denaturation (3,11,13). According to Webb, cryoconcentration can be subdivided into two types: amorphous phase and bulk-scale/progressive freeze-concentration (7). The *amorphous phase freeze-concentration* happens on the microscopic scale and is based on the unavoidable dehydration of the amorphous phase when water molecules form ice crystals (Fig. 1a). Freeze-concentrated solution (FCS) becomes trapped into micron-sized channel-like spaces in between the ice crystals.

When cooling the bulk to below 0°C FCS usually freezes at lower temperatures than water or stays liquid until trespassing its glass transition temperature T_g_’. The main focus of this work, though, is on *bulk-scale/progressive freeze-concentration* which happens on the macroscopic, centimeter scale (14). Solutes are progressively pushed into a certain direction by the growing ice front and hence are concentrated while freezing the bulk solution (Fig. 1b). One of the determining factors for bulk-scale freeze-concentration is the heat transfer in and out of the container. Macroscopic cryoconcentration is often identified as a cause of possible degradation of the protein (7,10,15,16). The change of chemical milieu in solution and limitation of mobility after freezing can cause the protein to assemble monomeric units and form dimers or trimers. These protein oligomers are referred to as “high-molecular weight species” (10) or simply as aggregates. Since aggregates in protein therapeutics can potentially induce immunogenicity (17,18) in the patients, the amount of permitted aggregates in a drug is strictly regulated and kept to an absolute minimum.

Size-exclusion chromatography (SEC) is an analytical method widely used in the pharmaceutical industry to detect and quantify the number of aggregates in monoclonal antibody (mAb) formulations. However, it has its restrictions, like the size range limitation to so-called soluble aggregates (19) or the increase in the level of soluble aggregates promoted by the preparation method (20). Most freeze/thaw (FT) studies are based on small-scale experiments (21), since experimenting with larger volumes is rather expensive and time-consuming (22). Most data published deal with FT behavior of solutions containing only solutes or basic proteins (*e.g.*, lactate dehydrogenase, bovine serum albumin). By contrast, in the production process of drug substances, pharmaceutical industries usually work with larger volumes in order to save time, container material and space, both during storage and transportation. Data retrieved from large-scale experiments are very scarce, and a clear need for study of freezing at the large scale exists. In our work, we, therefore, exclusively focus on the freezing and thawing of a specific in-house IgG_1_ mAb formulation in either 250-ml or 2-L PET-G bottles (Nalgene Thermo Scientific, USA). It is the aim of this work to investigate cryoconcentration effects after freezing and study the distribution of aggregates in the bottle. The focus in this work is on different freezing protocols, including different bottle volumes and bottle positions as well as different cooling rates and storage temperatures. In the future, data retrieved in this study can help to create a prediction model that will save time and costs, and accordingly enable more flexibility for manufacturing, with the goal to reduce the number of aggregates by providing guidance for optimal FT rates and formulation.

## MATERIALS AND METHODS

### Freezing Protocols for Bottles

**Fig. 2:**
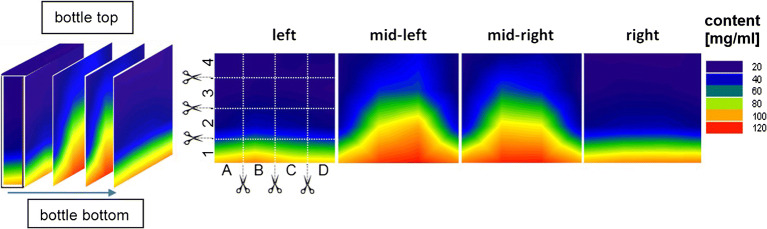
Schematic depiction of the cutting procedure of frozen bottles

For this study, nine PET-G bottles (Thermo ScientificTM, NalgeneTM, Waltham, Massachusetts, USA) were filled with 200 ml of an in-house IgG_1_ mAb solution, and five additional PET-G bottles were filled with 1.8 L. The mAbs are formulated in a 50.4-mM sodium citrate buffer with 160 mM trehalose. These bottles were frozen by using various protocols starting from room temperature (RT) (see, Table I). The protocols differ in terms of the volume of the bottle, the position of the bottle during freezing, the cooling protocol, and the storage temperature. The different cooling protocols include initial cooling to either − 5°C, − 8°C, − 20°C, − 80°C, − 130°C, or − 196°C. The storage temperatures are − 20°C, − 40°C, or − 80°C. As a frozen bulk each bottle was cut into 64 cubes (14 × 14 × 14 mm in size) by a benchtop metal saw (MACC, Schio, Italy) in the manner depicted in Fig. 2. First, the bulk formulation was cut into slices; each slice was cut into stripes which were again cut into cubes. Each cube was placed into a 50-ml tube (Sarstedt, Nuembrecht, Germany) and thawed to 2–8°C overnight.

**Table I:** Freezing Profiles/Storage Conditions of the mAb Formulation in PET-G Bottles of 250 ml (No. 1–9) and 2 L (No. 11–15)

Bottle number	Temperature profile*	Cooling technique	Position	Storage	
				Temperature	Time
250-ml bottles					
1	From RT to − 20°C holding for 48 h, then to − 40°C	Celsius®-S^3^ system^1^	Upright	− 40°C	12 days
2	From RT to − 20°C	Cold storage room	Upright	− 20°C	3 days
3	From RT to − 80°C	Blastfreezer^2^	Upright	− 80°C	12 h
4	From RT to − 80°C	Blastfreezer^2^	60° inclined	− 80°C	8 days
5	From RT to − 80°C	Blastfreezer^2^	60° inclined	− 20°C	18 days
6	From RT to − 130°C	Cryo-chamber^3^	Upright	− 80°C	1 day
7	From RT to − 196°C	Dipped into LN_2_	Upright	− 80°C	6 days
8	From RT to − 5°C for 2 h and then to − 130°C	Cryo-chamber^3^	Upright	− 80°C	12 days
9	From RT to − 8°C for 5 days, after to − 9°C for 5 days, after to − 15°C for 4 days	Celsius®-S^3^ system^1^	Upright	− 20°C	14 days
2-L bottles					
11	From RT to − 80°C	Blast freezer^2^	Upright	− 80°C	1 day
12	From RT to − 80°C	Blast freezer^2^	60° inclined	− 80°C	1 day
13	From RT to − 80°C	Blast freezer^2^	60° inclined	− 80°C	1 day
14	From RT to − 80°C	Blast freezer^2^	60° inclined	− 80°C	1 day
15	From RT to − 80°C	Blast freezer^2^	Upright	− 80°C	1 day

^1^Celsius®-S^3^ system (Sartorius-Stedim Biotech, Germany) is a small-volume controlled freeze-thaw system that allows *in vivo* temperature monitoring of samples placed in a cryochamber

^2^A blast freezer (Thalheimer Kühlung, Germany) works as a usual freezer with the difference that a constantly flowing cold airstream from the bottom is carrying away the heat produced in the chamber

^3^Temperature control in the cryochamber (Consarctic, Germany) works with liquid nitrogen (− 196°C) as a coolant and allows programmable temperature profiles

*Indicated temperatures refer to the set-point temperatures in the particular cooling system which differs from the sample temperatures

### Determination of Protein Concentration and Number of Aggregates

Liquid mAb solution was sterile-filtered before determining the protein content by using UV-Vis spectroscopy (SoloVPE System by C Technologies, Inc., Bridgewater, New Jersey, USA). The local relative concentration C/C_0_ is determined as the measured concentration divided by the initial pre-freeze concentration, C_0_ = 56 mg/ml. The number of aggregates was detected by size-exclusion high-performance liquid chromatography (Agilent Technologies, Santa Clara, California, USA). Before measurement, each sample was diluted. The SEC was conducted using a TSKgel G3000SWXL column (Tosoh Bioscience LLC, King of Prussia, Pennsylvania, USA) in a 150-mM potassium phosphate buffer at a flow rate of 0.4 ml/min. For each sample run, 10 μl were injected and detected by a UV detector. SEC was validated according to guidelines for the pharmaceutical industry (showing a precision and limit of quantitation of 0.10% and a repeatability of 6% (= relative standard deviation).

Contour maps of aggregate distribution are shown relative to the average fraction of aggregates in the entire bottle. In order to determine protein and aggregate distribution upon volume upscaling, five 2-L PET-G bottles (Thermo ScientificTM, NalgeneTM, Waltham, Massachusetts, USA) were filled with 1800 ml of the same mAb formulation and cooled to − 80°C in the blast freezer. After freezing, the bottles were cut into approximately 190 cubes (5 × 5 × 7 + 10 to 15) 2.1 × 2.1 × 2.1 cm in size. In order to determine protein concentration, affinity liquid chromatography (ALC) was done using a protein A-coated column (Applied Biosystems, Foster City, California, USA). The number of aggregates was analyzed in the same manner as for the 250-ml PET-G bottles.

### Optical Cryomicroscopy on Droplets

Optical cryomicroscope BX-51 (Olympus Corporation, Japan) was used to image the distribution of solution and ice crystals. A droplet (approx. 0.5 μl) of 50.4-mM sodium citrate buffer (mAb buffer without trehalose) was placed on an object plate and cooled in a temperature controlled cryostage LTS420 (Linkam Scientific Instruments, UK) using liquid nitrogen as a cooling medium. Samples were cooled to − 70°C at different rates between 0.5 and 5°C/min. Images were taken at − 70°C using a CCD camera after calibrating a ULWD 5×−50× objective (Olympus Corporation, Japan).

## RESULTS AND DISCUSSION

### Cooling Rate Dependence of Ice/Solution Distribution in Droplets

**Fig. 3:**
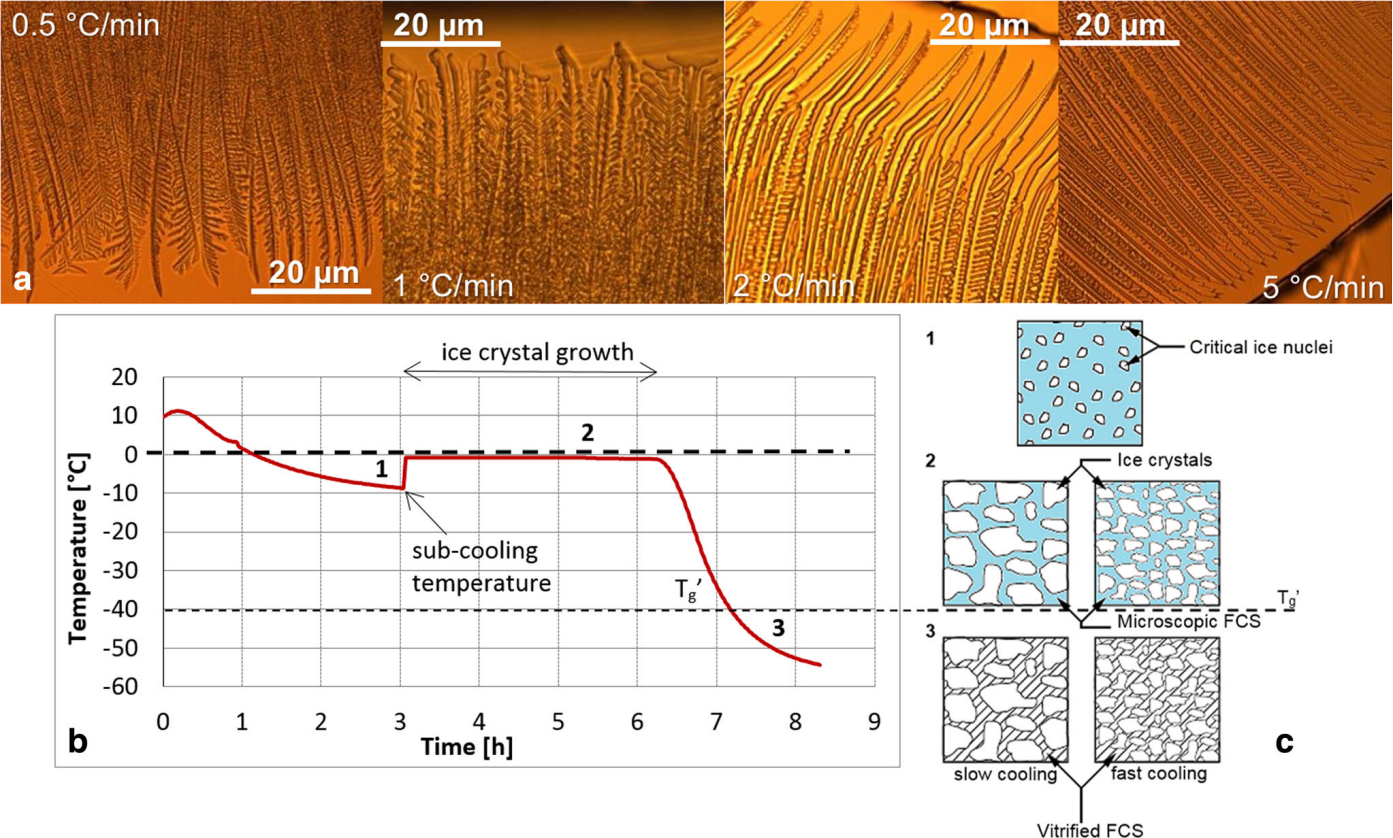
**a** OCM images of a droplet-containing sodium citrate buffer recorded at − 70°C after being cooled at different rates, **b** temperature profile of aqueous solution during cooling to a set-point temperature of − 60°C measured in the center of the bottle with a thermocouple, and **c** illustration of ice crystal growth in solution during freezing at slow and fast cooling. Adapted from original publication (23)

We have investigated the freezing process of sodium citrate buffer (without trehalose) and the protein formulation **(**see Supplementary Material) with optical cryomicroscopy (OCM). The ice crystal morphology of sodium citrate buffer after freezing shows clear differences depending on the cooling rate. At − 70°C, OCM images of the droplet after applying slow cooling rates (0.5 and 1 °C/min) reveal highly branched ice dendrites that partially overlap each other (Fig. 3a, left images).

By contrast, faster cooling (2 and 5°C/min) results in long needles with less branches and noticeably larger interdendritic space (Fig. 3a, right images). The cooling rate does not only affect the morphology of ice crystals itself but also has an impact on the distribution of solute (3,13,23). Generally, slow cooling favors the development of fewer but larger ice crystals, whereas fast cooling favors the formation of more but smaller ice crystals (Fig. 3c). Thus, fast cooling generates a larger ice-liquid interface, at which protein may be adsorbed and unfolded (11,13). Furthermore, ice crystal growth velocity (24) is typically larger for fast cooling. This is a consequence of the subcooling temperature being lower for fast cooling (13) (Fig. 3b for a definition of the subcooling temperature). In Fig. 3c, the ice crystals all emerge from a single nucleation site only near the center of the droplet. As a consequence, there are two types of FCS: one that is interwoven between the ice crystals and one that is pushed forward by the growing ice front. Thus, subcooling temperature and crystal growth rate might also be key factors for the freezing of large amounts of the solution in bottles.

### Distributional Changes of Protein

**Fig. 4:**
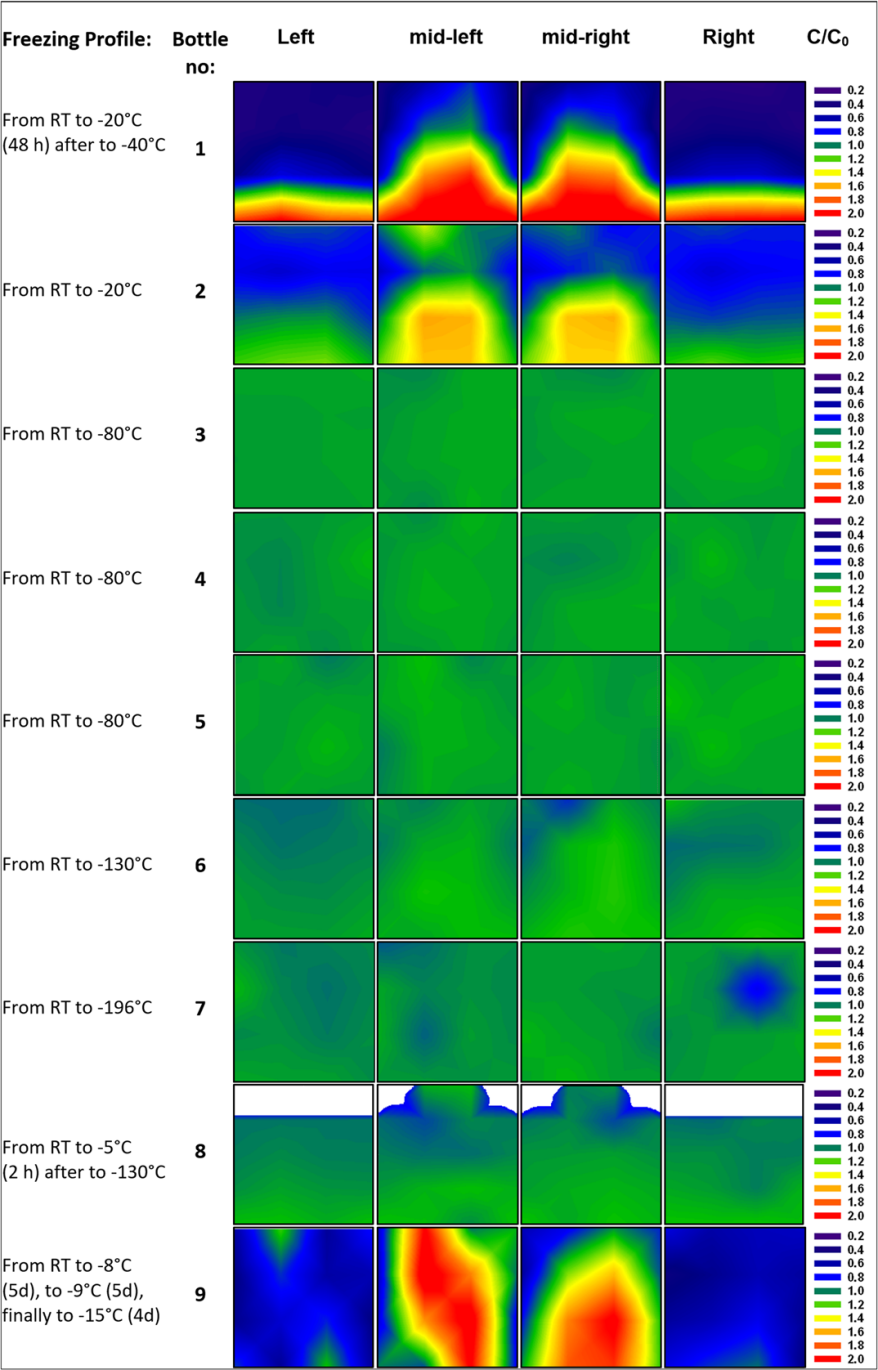
Contour maps for relative concentration [C/C_0_] of mAb in the frozen 250-ml PET-G bottle, showing the distribution of the protein in the four slices of the bulk. White areas represent headspace above the surface

Figure 4 shows contour maps of the relative concentration [C/C_o_] of mAb in the nine 250-ml bottles frozen with different freezing procedures as determined from the UV-Vis spectra. It is immediately evident that bottles 1, 2, and 9 show protein hot spots, whereas all other (bottles 3–8) show a rather uniform spatial distribution of protein. Bottles 3–7 are directly cooled to temperatures of − 80°C (or below). Bottle 8 is first supercooled to − 5°C (without freezing, see temperature profiles in Supplementary Material) and then directly cooled to − 130°C. Bottles 1 and 2, by contrast, are cooled to − 20°C only, whereas for bottle 9 freezing is induced at − 8°C. Thus, fast cooling to − 80°C or below results in a rather homogeneous distribution of protein, whereas staying for longer periods of time at − 20°C or above in the course of freezing results in hot spots. Heating the frozen solution back to − 20°C and storing it there for 18 days, however, does not result in hot spots—as is evidenced from bottle 5. Thus, it is the freezing process itself that generates the hot spots, whereas they are not induced in the course of storage. Storage for 18 days at − 20°C does not significantly affect the relative concentration of protein. Bottle 5, which was stored in this way, shows a very similar distribution of protein as bottle 4, which was stored at − 80°C (Fig. 4). The highest relative concentration (RC) of 2.4 was found at the center of bottle 1 which was cooled to and stored at − 20°C for 48 h in the upright position. Similarly, bottle 9 shows a maximum RC of 2.19. This one was cooled by the slowest procedure, namely staying for 5 days at − 8°C, followed by additional isothermal steps at − 9 and − 15°C using the Celsius®-S^3^ System. The highest RCs are found along the center line of the bottle starting from the center of the bottom layer lancing to the center of the top layer suggesting that the freezing-fronts originating from the side surface of the bottle pushed the protein towards the center. Pruppacher showed that the growth rate of ice crystals increases rapidly, approximately quadratically with the subcooling (24). According to his data, a subcooling temperature of − 4°C, − 5°C, and − 5.5°C (like in bottle 1, 8, and 9) corresponds to an ice crystal growth of 8 mm/s, 12 mm/s, and 15 mm/s (24). Faster growth rates of ice crystals then also result in shorter freezing plateaus (25,26), as evident in the accompanying temperature profiles (Supplementary Material). When the formed ice front reaches the center of the bottle, the remaining unfrozen solution is pushed up to the top of the surface where it finally freezes in form of an iceberg. When slower cooling rates are applied, like for instance bottle 1, the side slices hardly contain protein (RC ≈ 0.5) which indicates the direction of proliferation of the dendritic ice crystal front. The accumulation of solutes and protein in the lower, center half of the bottle can be explained by temperature-gradients in the bottle that lead to a convective downward flow of solution during cooling, and hence to macrocryoconcentration of the lower, central regions (27). Besides of the hot spots, there is still some protein (RC = 0.5–1) remaining in the rest of the bulk which indicates that protein is entrapped in the interdendritic spaces during freezing.

This entrapment is observed in all bottles, and the dominant mechanism for bottles 3–8. Hardly any fluctuation of relative protein concentration is encountered, and we surmise this to be due to the formation of a lot of small ice crystals that trap the protein and limit their transport away from the interface (21). Therefore, the results support the statement that faster freezing (by cooling as fast as possible in the blast freezer or with liquid nitrogen to − 80°C and below) hinders macrocryoconcentration (resulting in hot spots), but results in microcryoconcentration. An important factor to consider during freezing a bulk is the removal of the released latent heat. In the blast freezer, the heat is efficiently removed by the − 80°C cold air stream from the bottom which leads to faster cooling and consequently could be a reason for the more homogeneous distribution of the protein in the bottle. Protein being trapped in the small veins of the numerously newly formed ice crystals throughout the bulk is the reason for the well-distributed protein in bottles 3–8.

**Fig. 5:**
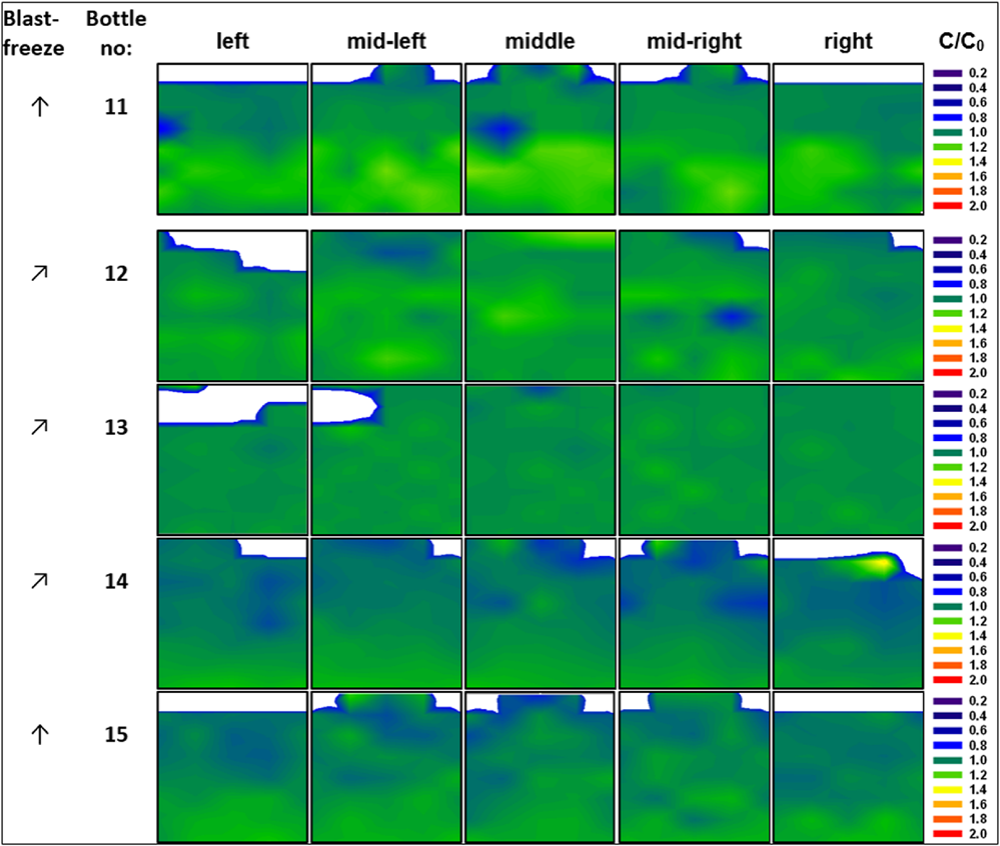
Contour maps for relative concentration [C/C_0_] of mAb in the 2-L PET-G bottle after freezing in the blast freezer to − 80°C. Arrows indicate positioning of the bottle in either an upright position (↑) or at a 60° angle (↗). White areas represent headspace above the surface

The heat removal during cooling is mainly taking place at the side walls of the bottle, and the surface to volume ratio affects the cooling rates and consequently also the velocity of the ice front. It is, thus, conceivable that larger volumes behave differently. In order to assess whether the cooling rates in the blast freezer are also sufficient to freeze larger volumes without significant macrocryoconcentration, we have also studied 2-L bottles. This is indeed the case, as demonstrated in Fig. 5. Protein distribution in 2-L bottles after freezing in the blast freezer is quite homogeneous and similar to the distribution pattern of the 250-ml bottles 3–8. Temperature profiles of 2-L bottles (Supplementary Material) indicate that the last point to freeze is the upper surface/iceberg, which might explain some spots of slightly increased protein concentration near the surface (*e.g.*, RC~1.4 in bottle 14).

Summing up, cooling at − 20°C (bottle 1 and bottle 2) as well as pre-cooling at − 8°C and freezing at − 9°C for 5 days and − 15°C for 4 days prior to storage at − 20°C (bottle 9) lead to an increased macrocryoconcentration in the bottle. However, if the bottle is cooled directly to − 80°C or lower macrocryoconcentration is prevented (bottles 3–7). Pre-cooling at − 5°C for 2 h without initiating the freezing process has no detrimental impact on homogeneous distribution of protein (bottle 8). Thus, homogeneous distribution of protein can be ensured by cooling the bottles rapidly to − 80°C rather than staying for many hours at − 15 or − 20°C. Larger volumes can still be frozen homogeneously, even though it is evident that smaller volumes lead to a slightly smaller deviation from RC = 1.

### Distributional Change of Number of Aggregates

**Fig. 6:**
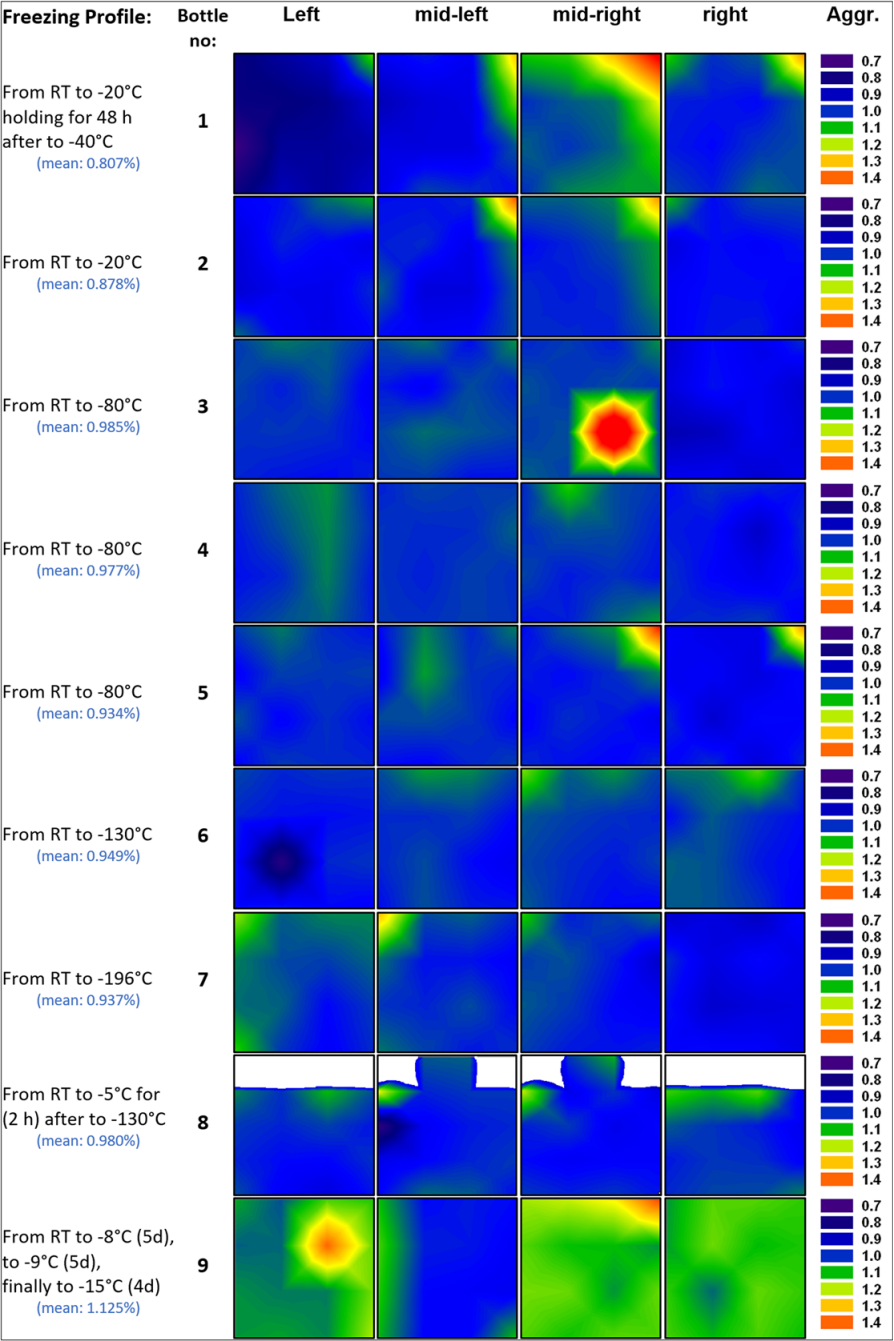
Contour maps for the mAb aggregates [values are given relative to the average fraction in the bottle mentioned in the left column in brackets] in the frozen 250-ml PET-G bottle, showing the distribution of the number of aggregates in the four slices of the bulk. White areas represent headspace above the surface

Contour maps of aggregate distribution as determined from SEC in the 250-ml bottles are shown in Fig. 6. Averaged over the whole bottle, the aggregate mean is determined to be 0.93–0.98% for bottles 3–8, *i.e.*, the ones which show a rather uniform protein concentration. Bottle 1 and bottle 2 show only 0.80 and 0.88%, whereas bottle 9 shows 1.13%. The distribution is rather narrow for bottles 3–8, but more widespread for bottles 1, 2, and 9. Hot spots exceeding 1.5% can be found, *e.g.*, in bottle 1 near the top, whereas it is 0.7% or less near the sidewalls. By contrast, bottles 3–8 have barely any hot spots,^1^ but still a slightly higher fraction of aggregates at the top surface, mostly in one corner or centered like in bottle 6 and 8. This can be traced back to the fact that the iceberg is the last part to freeze, resulting from the sudden increase of volume by roughly 9% (28). Examination of the top layer and the iceberg with the unaided eye shows morphological differences to the frozen bulk. The top layer appears to be porous, brittle, and more opaque due to entrapped air bubbles. It has to be mentioned that the iceberg-like structure on top of the surface of the frozen 250-ml bottles was not analyzed separately (except bottle 8) but rather mixed with the cube cut from the layer underneath, *i.e.*, the iceberg structure might contain a larger number of aggregates.

The fast trespassing of T_g_’ (T_g_’ of trehalose around − 30°C (29) and T_g_’ of protein solution between − 30 and − 35°C (Supplementary Material) by cooling directly to − 80°C in bottles 3–8 limits the transport of protein by convection and diffusion in the microchannels in between the ice crystals. Previous X-ray diffraction measurements show no signs for crystalline trehalose in protein formulation during cooling to − 80°C nor during storage at − 80 and − 20°C (Supplementary Material).

The fact that the average number of aggregates in bottles 1 and 2 is lower, but in bottle 9 higher than in bottles 3–8 (Fig. 6) shows that there is no direct correlation between increased protein content and high number of aggregates. Comparing the location of the hot spots in Fig. 4 and Fig. 6 leads to the same conclusion that protein concentration and number of aggregates are not correlated, while most aggregates are at the top surface and at the left sidewall of the bottle, the highest protein concentrations are found at the centerline of the bottle.

**Fig. 7:**
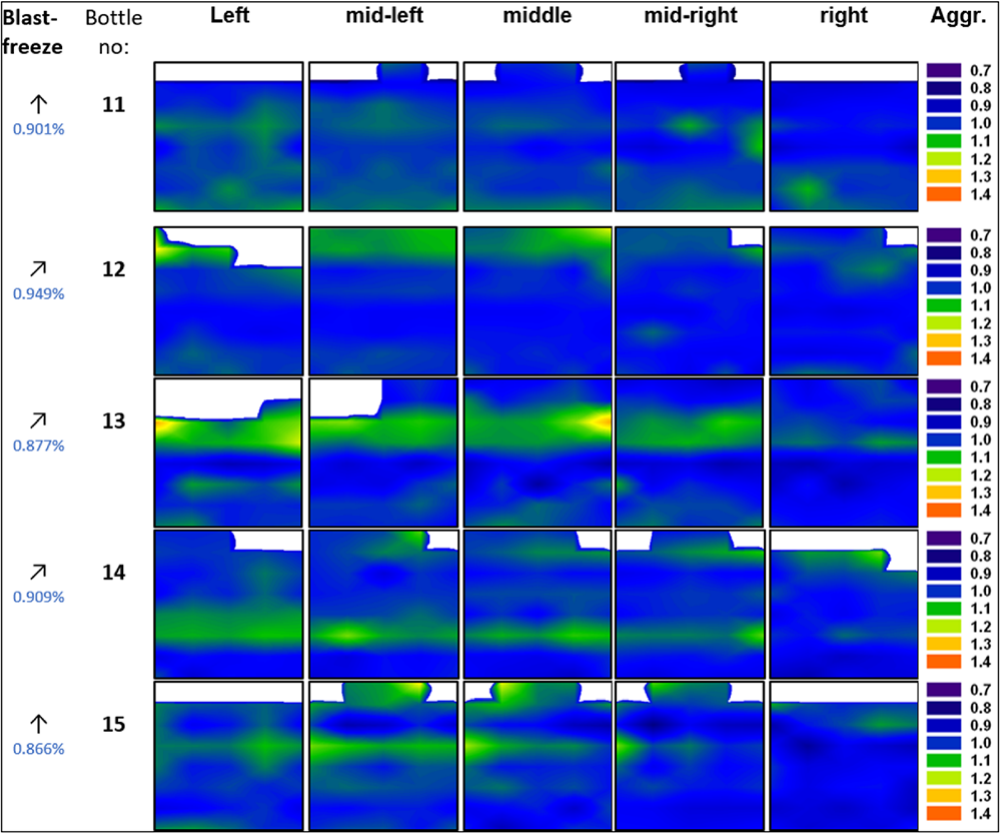
Contour maps for the mAb aggregates (values are given relative to the average fraction in the bottle mentioned in the left column) in the 2-L PET-G bottle frozen in the blast freezer to − 80°C. Arrows indicate positioning of the bottle in either an upright position (↑) or at a 60° angle (↗). White areas represent headspace above the surface

In the frozen 2-L bottles, the average number of aggregates is between 0.87 and 0.95%, similar to the value found for the 250-ml bottles 3–8. Thus, the size of the bottle does not seem to have a significant influence on aggregate formation. However, the distribution of aggregates is less homogeneous than for 250-ml bottles (Fig. 7). Except for bottle 11, all 2-L bottles reveal an accumulation of aggregates (increase of up to 0.25%) near the top surface and in the iceberg.

**Fig. 8:**
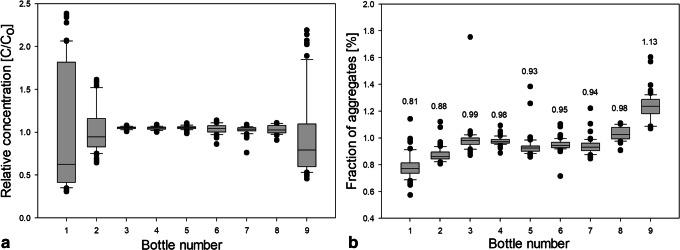
Box plot of **a** relative mAb concentration and **b** fraction of aggregates in 250-ml PET-G bottles after applying different freezing profiles. Numbers above boxes indicate the mean value

**Fig. 9:**
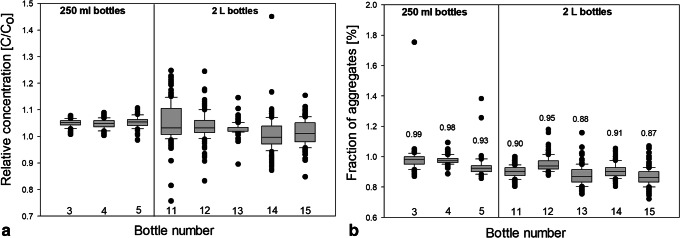
Box plot of **a** relative mAb concentration and **b** fraction of aggregates in 250-ml PET-G bottles and 2-L PET-G bottles after freezing in the blast freezer. Numbers above the box indicate the mean value

This leads to the question, which factors govern the aggregate numbers, *i.e.*, why bottle 9 has the highest average, whereas bottles 1 and 2 the lowest. In order to help answer this question, Figs. 8 and 9 show box plots for the protein concentration (panel a) and the number of aggregates (panel b) for 250-ml and 2-L bottles, respectively. The data are presented as a box which contains 50% of all measured values. The band inside the box represents the median. The ends of the whiskers mark underneath the 10th percentile and above the 90th percentile. All data not already within the whiskers are shown as black dots. It is immediately evident (Fig. 8a) that the protein concentration is highly inhomogeneous in bottles 1, 2, and 9. This translates into an inhomogeneous distribution of aggregates (Fig. 8b). Bottle 9 spent 5 days at − 8°C after freezing has occurred. At such high temperatures (clearly above T_g_’), protein diffusion in the microchannels might be the mechanism leading to the high number of aggregates that are spatially rather homogeneously distributed within bottle 9. Furthermore, concentrated protein in FCS can unfold on the ice-water interface and form aggregates during freezing (11,30). That is, the total ice-liquid interface area being larger in bottle 9 than in all other bottles may be at the origin of the large number of aggregates. Similarly, air entrapment which is released during thawing may have caused protein denaturation and aggregation on air-liquid interfaces (31,32).

That is, to avoid aggregates it needs to be avoided to keep the bottle at high temperatures *after* freezing. Keeping the bottle for long times at such temperatures *before* freezing, however, is not an issue. The low number of aggregates in bottle 8 testifies this statement. Bottles 1 and 2 were frozen by cooling to − 20°C and then staying there for 2 days or storing it at − 20°C. This results in high macrocryoconcentration (Fig. 4) and a low average number of aggregates, but lots of hot spots for aggregates (Fig. 6). In other words, keeping the bottle for long times at − 20°C after freezing, *i.e.*, above T_g_’, results in an inhomogeneous distribution of aggregates. We attribute this inhomogeneity in terms of aggregation to be a result of ice-liquid and/or air-liquid interfaces, *i.e.*, aggregation taking place in the viscous FCS pushed forward by the propagating ice front. Cooling to T < T_g_’ avoids this effect. However, microcryoconcentration and the FCS interwoven between ice crystals play a role for all freezing protocols.

Of all bottles, the ones cooled directly to − 80°C in the blast freezer (bottles 3–5) show the most homogeneous distribution of protein (thin box around a C/C_o_ value of 1 in Fig. 8a). Nonetheless, these three bottles show a slight increase of the mean value of aggregates compared to bottle 1 (Fig. 8b). A possible reason for a slight increase of aggregates in bottles frozen in the blast freezer or bottles cooled with liquid nitrogen could be based on microcryoconcentration in between the ice dendrites/microchannels. Freezing protein solution by using liquid nitrogen as a coolant (bottles 6–8) causes a slightly higher inhomogeneity of protein distribution. However, those bottles have shown no significant change of fraction of aggregates, compared to bottles frozen in the blast freezer.

Directly comparing 250-ml bottles with 2-L bottles frozen in the blast freezer, it can be noticed that 2-L bottles generally show a more inhomogeneous distribution of protein, as is evident from a two to four times bigger box in the box plot (Fig. 9a). The increase of inhomogeneity in 2-L bottles can be explained by the decrease of the specific surface which limits the heat convection to the surrounding. Also, an increased number of outliers can be noticed. This means that a volume increase leads to a higher chance of macrocryoconcentration in the bottle due to the fact that slower cooling facilitates the formation of large pockets with elevated protein concentration.

Despite of less homogeneous distribution of aggregates in the 2-L bottles, the mean values of aggregates prove to be very similar (Fig. 9b). Also, the scatter of the data points does not increase significantly (highest increase by a factor 1.5) when freezing formulation in 2-L scale. With other words, Fig. 9b demonstrates that upscaling does not cause an augmentation of aggregates. The increase of macrocryoconcentration seems to have no negative impact. This confirms the abovementioned idea that aggregate formation preferentially takes place at the propagating freezing front, *i.e.*, in the zone pushed forward by the growing ice crystals. In this scenario, the location of FCS depends on the ice front velocity and indirectly on the geometry of the bottle, *i.e.*, from which side area the freezing process starts (Supplementary Material). Whether the bottle is placed in an upright or tilted position in the blast freezer does not result in significant differences in terms of distribution of aggregates.

## CONCLUSION

In this work, we have investigated the freezing of a specific mAb formulation at the large-scale of 250 ml and 2 L, which has been a vast gap in literature so far. In particular, we focus on the effects of freeze-concentration on the homogeneity of the distribution of protein and on the number of aggregates within the bottle. These two properties are investigated locally by cutting the frozen bottles in dozens of small cubes. We find that a rather homogeneous distribution of protein is reached by cooling directly to − 80°C, *i.e.*, by rapidly trespassing T_g_’ after initial freezing. Staying for extended periods in the supercooled liquid state, *e.g.*, at − 5°C, is not adverse. However, staying for extended periods at similar temperatures after freezing, *e.g.*, − 8°C, results in a large inhomogeneity and several hot spots. Similarly, staying for days after freezing at − 20°C results in a large number of hot spots. The hot spots are mostly located in the bottom center part of the bottle—which is explained by ice crystals growing inward from the side walls and macrocryoconcentration. By contrast, storage at − 20°C after cooling directly to − 80°C results in a homogeneous distribution of protein. That is, the freezing process itself, not the storage temperature is the key.

Interestingly, we find that aggregates do not necessarily form in regions of high macrocryoconcentration, *i.e.*, in the “hot spots.” By contrast to hot spots of protein concentration, the hot spots of aggregates are typically observed at the top part of the bottle. We explain this fact by looking into macro- and microcryoconcentration effects which include ice crystal growth rates and size/amount of ice crystals formed in the bulk depending on cooling rates. Slower cooling rates (≤ 1°C/min) result in highly branched ice dendrites but fewer ice crystals contrary to faster rates (≥ 2°C/min) leading to formation of a lot of small ice crystals entrapping the solutes and protein. The high microcryoconcentration of protein in the FCS (up to RC ≈ 10) and the larger specific surface area of the ice crystals (area of unfolding of protein) are two possible reasons for the higher fraction of aggregates in bottles cooled at faster rates.

Accordingly, our work shows that microcryoconcentration is at the origin of aggregate formation but not macrocryoconcentration. The upscaling from 250 ml to 2 L results in an up to fourfold increase of macrocryoconcentration but, however, a slight decrease of the total number of aggregates in the whole bulk. The highest number of aggregates is mostly located at the last point to freeze which depending on the freezing procedure is located either at the center/bottom or at the upper surface layer of the bottle or both.

As a strategy to avoid aggregates, it is thus advisable to grow large ice crystals rather than many small ice crystals of large surface area. This goal can be reached by avoiding deep supercooling and initiating the freezing process as close as possible to 0°C. One strategy to achieve that goal, which was not tested in the present work, would then be to externally add ice crystals once the solution is below 0°C and avoid subcooling through heterogeneous nucleation. Another option would be the initiation of freezing by an external compressional pulse, *i.e.*, shaking below 0°C. After initiation of freezing, fast cooling to temperatures well below T_g_’ will prevent inhomogeneous protein distribution. Keeping the solution for an extended period of time at the nucleating temperature/onset temperature of freezing > T_g_’ (*e.g.*, − 20°C) should be avoided.

## Electronic supplementary material

ESM 1 (DOCX 5.76 mb)

## Footnotes

1 In bottle 3, the one data point with an excessively high number of aggregates (1.75%) is considered to be an outlier with no further consideration.
