# Supplementary material for: Distribution of Protein Content and Number of Aggregates in Monoclonal Antibody Formulation After Large-Scale Freezing
Source: AAPS PharmSciTech. 2019 Jan 10;20(2):72. doi: 10.1208/s12249-018-1281-z (PMC6373418; doi:10.1208/s12249-018-1281-z)
Supplement: Supplementary file 1 — (DOCX 5.76 mb) [file 12249_2018_1281_MOESM1_ESM.docx]

**Distribution of Protein Content and Number of Aggregates in Monoclonal Antibody Formulation after Large-Scale Freezing**

**Astrid Hauptmann^1^, Georg Hoelzl^2^ and Thomas Loerting^1*^**

**SUPPLEMENTARY MATERIAL**

***Temperature profiles during freezing:***

Temperature profiles support understanding of the freezing process in a liquid solution. For instance, the subcooling temperature and the length of the freezing plateau can be extracted. In our case we were measuring temperature profiles in order to locate where the freezing process in the bottle starts and in which direction the freezing front moves. The 2-L bottles show a similar distribution of protein (see Fig. 4 and Fig. 5) and number of aggregates (see Fig. 6 and Fig. 7) after freezing as the 250-ml bottles. Thus, we assume that the findings in 2-L bottles can be transferred to 250-ml bottles.

To determine temperature profiles we have equipped 2-L PET-G bottles with 10 thermocouples at the locations indicated in the insets of Fig. S.1 and Fig. S.2. When the bottle is placed in an upright position, during freezing to – 80°C the first thermocouples detecting the freezing plateau are the ones at the corners of the base area (TC 1, 2 and 3). This means that ice is formed first at the corners of the bottom area of the bottle. Next, the freezing process at the center of the base area is finished (TC 5) and temperature drops, however, at a lower rate than at the corners of the bottom area. Nonetheless, even though the temperature measured at the side surface of the bottle (TC 4) exits the freezing plateau later than at the center base area it cools down a lot faster. After about an hour – 60°C has been reached at the corners of the bottom, – 35°C at the sidewall and in the center of the bottom area, but 0°C everywhere else. That is, the freezing front moves both upwards and inwards. Thermocouples placed at the center line of the bottle (TC 5, 6, 7, 8, 10) consecutively exit the freezing plateau bottom-up and reach the set-point-end-temperature.


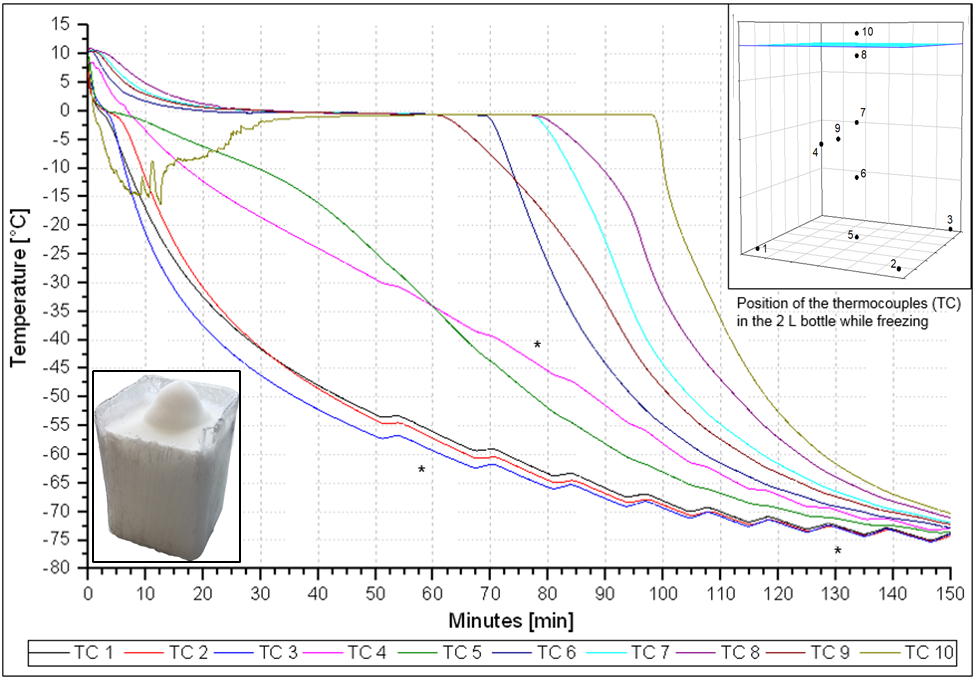


**Fig. S.1:** Temperature profiles of a 2-L bottle filled with protein solution frozen in the blast-freezer to – 75°C in the upright position. * Fluctuations in the temperature curves result from the automated switching of the refrigeration compressor in the blast-freezer.

Due to the expansion of ice the solution in the center of the bottle evades and forms an “iceberg” on top of the surface line. Temperatures measured above the surface (TC 10) fluctuate due to the fact that the temperature of the cold air stream is recorded first. Later, as soon as the “water/ice” level has raised enough the thermocouple penetrates the liquid and starts recording the temperature of the freezing solution. This temperature curve (olive-green line in Fig. S.1) is, by 20 min, the last one dropping to sub-zero temperatures, proving that position 10 is the last point to freeze after a total time of approximately 110 min.

In short, the freezing process of a 2-L bottle in the upright position in the blast freezer starts from the bottom. A few minutes later the sidewalls (pink line in Fig. S.1) form a freezing front. Ice crystal growth is starting from the sidewalls and from the bottom proliferating into the center. The liquid core in the bulk with 0°C temperature is then pushed out of the bulk in the course of the final step of freezing, leaving an iceberg structure on top of the surface to be the last point to freeze (olive-green line in Fig. S.1).

When tilting the bottle on one corner (position 2 in Fig. S.2) at a 60° angle the freezing process starts at the two corners of the bottom area (position 1 and 2) and the two sidewalls (α and β in Fig. S.2, inlet), as they are the most directly exposed to the – 80°C cold air stream (see Fig. S.2). At the opposite corner (position 3) of position 2 the freezing takes place about 6 minutes later. At almost the same time (about 1 minute later) TC 4 is recording a freezing plateau which shows that the freezing process is proceeding at the other two walls of the bottle. From that point on the freezing process in the 60° tilted bottle follows a similar pattern as the bottle frozen in the upright position. Differences can be seen on TC 10 which first penetrates the bulk after 75 min compared to 40 min when in upright position. However, the last point finishing the freezing process is located just underneath the “water/ice” level (position 9 in Fig. S.2).


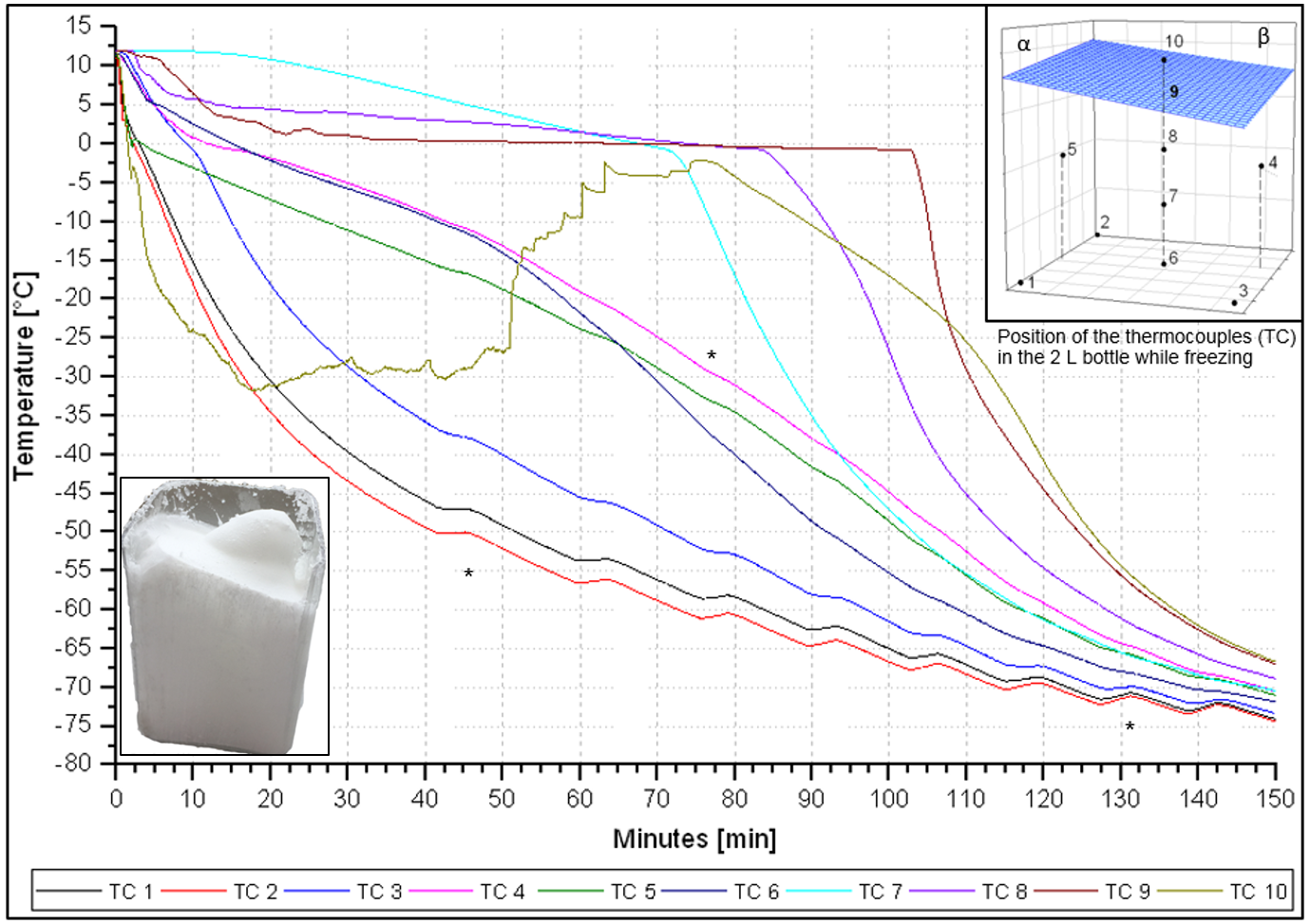


**Fig. S.2:** Temperature profiles of a 2-L bottle filled with protein solution frozen in the blast freezer to – 75°C in the 60° tilted position. * Fluctuations in the temperature curves result from the automated switching of the refrigeration compressor in the blast freezer.

Fig. S.3 shows temperature profiles of 250-ml bottles 1, 7, 8 and 9. The arrows in Fig. S.3 indicate the times at which the temperature suddenly increases, which is a result of the latent heat released by the freezing event. The temperature jump after the freezing onset is followed by a freezing plateau, which indicates the time-span between first and last freezing in the bottle. The freezing process in bottle 1 starts after reaching a subcooling temperature of – 4°C and takes about 30 minutes. When the bottle is directly plunged into liquid nitrogen (see Fig. S.3, B) no freezing plateau can be seen, meaning that the temperature in the center of the bottles immediately drops to lower temperatures. Interestingly, no subcooling of the sample was observed. However, if the bottle is pre-cooled to – 5°C for two hours the samples’ temperature goes into a freezing plateau for about 20 minutes (see Fig. S.3, C). Bottle 9 has remained in the subcooling liquid state at – 5.5°C for 5 days before freezing of the bulk was initiated when the set-point temperature in the system dropped to – 9°C (see Fig. S.3, D). The freezing plateau at 0°C measured at the center of the bottle lasted approximately 15 minutes, about 5 minutes shorter than in bottle 8.

**
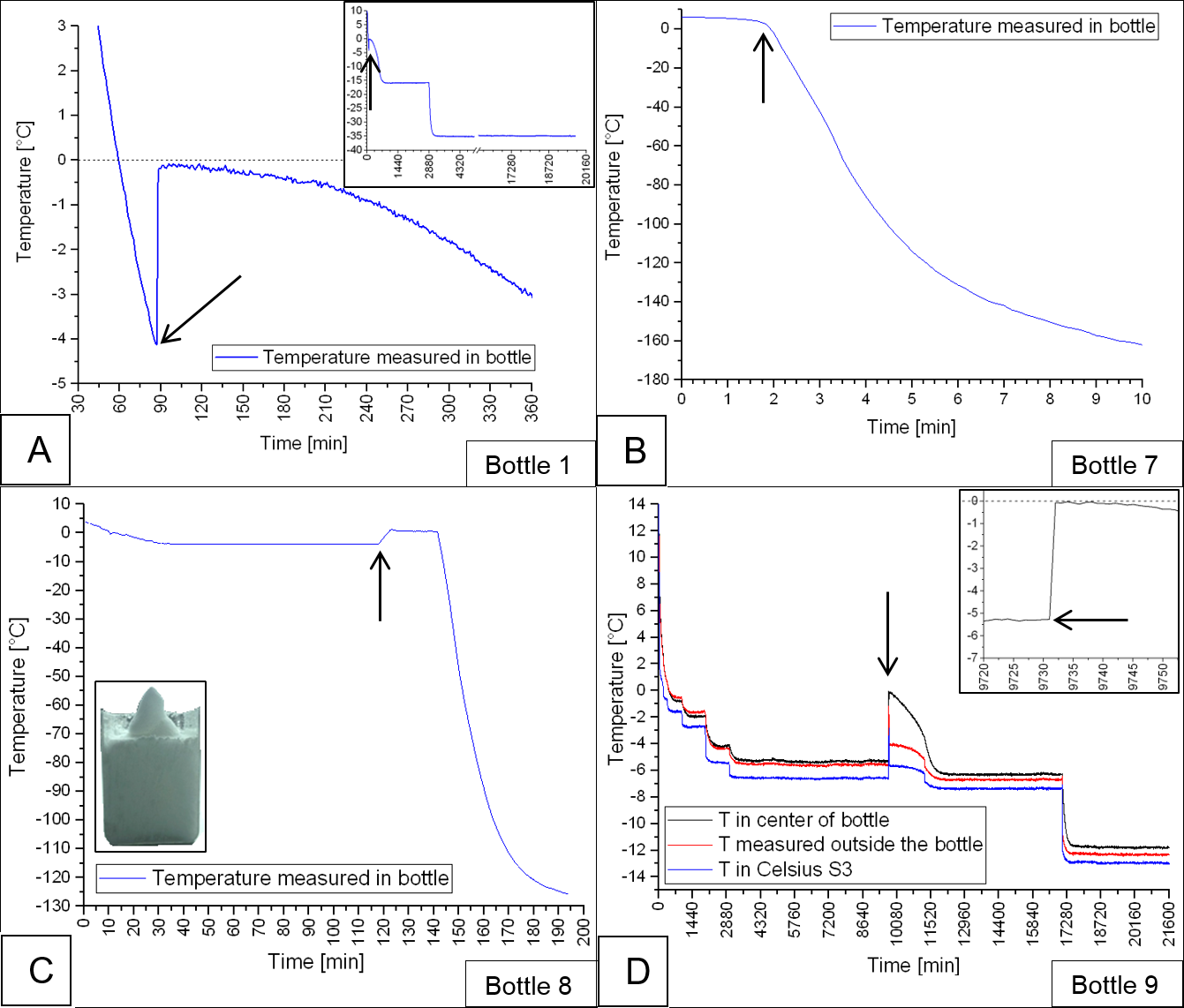
Fig. S.3:** Temperature profiles of 250-ml bottles filled with protein solution frozen (A) in the Celsius-S3® System, (B) by plunging directly into LN_2_, (C) in the cryo-chamber and (D) in the Celsius®-S^3^ system. The onset of freezing is marked by an arrow ↑.

A correlation can be seen that the lower the subcooling temperature before freezing, the shorter the freezing plateau is. Subcooling temperatures of – 4°C, – 5°C and – 5.5°C are followed by a freezing process taking 30 min, 20 min and 15 min, respectively.

***X-ray powder diffraction (XRD) measurements:***

In order to rule out trehalose crystallization as a possible cause for aggregate formation XRD measurements were conducted on 160 mM trehalose solution and on the protein formulation.

First, solid pure trehalose was measured at ambient temperature to get a reference diffractogram of crystalline trehalose (see Fig. S.4, top trace). Then, 160 mM trehalose solution was cooled to ≈ 211 K starting from ambient temperature. While cooling at rates of 5±2 K/min eight diffractograms were recorded (see Fig. S.4). On the one hand, it is clearly evident that Bragg peaks pertaining to hexagonal ice appear at ≈ 253 K (marked by * in Fig. S.4). On the other hand trehalose Bragg peaks are not seen in Fig. S.4, grey area.

The 160 mM trehalose/water mixture (see Fig. S.5, grey trace) and protein formulation (see Fig. S.5, blue trace) were directly cooled to 193 K at a rate of 5±2 K/min. In order to reach a very high S/N ratio X-ray data were collected for more than 20 hours in the range 8-22°. In spite of the very low noise in the measurement, there is no indication of any signal from crystalline trehalose, but rather very intense Bragg peaks from hexagonal ice. The long measurements were done to ensure that even small amounts of crystalline trehalose in the micron-sized veins after freeze-concentration would be seen in the diffractograms. Similarly, a XRD measurement with protein formulation at 253 K was conducted after filling the sample chamber with argon, which resulted in intensity loss of the ice Bragg peaks, but no crystallization of trehalose (see Fig. S.6, red trace).





**Fig. S.4:** XRD measurements of pure trehalose (first trace) and 160 mM trehalose solution upon cooling from ambient to 211 K at cooling rates of 5±2 K/min (lower traces). The * indicates Bragg peaks of hexagonal ice.





**Fig. S.5:** XRD measurements of 160 mM trehalose solution at 193 K (grey trace) and protein solution at 193 K (blue trace) and 253 K (green trace). All three diffractograms were measured for over 20 hours.

In a second attempt, the temperature was kept constant at 253 K for over 20 hours while diffractograms were recorded in an evacuated chamber (see Fig. S.5 and S.6, green trace). The vacuum causes ice to sublime, leading to drying-out of the sample (see Fig. S.6, inlet). The dried sample at 253 K shows a very broad halo peak found at angles of 18-19° indicating the formation of glassy trehalose (see Fig. S.6, green trace). In comparison, the halo-maximum of glassy water is found at 24°. That is, glassy trehalose forms even at the highest concentrations, prior to the last parts of ice subliming.





**Fig. S.6**: XRD measurements of protein solution containing 160 mM trehalose at 253 K. Samples were measured over 20 hours in an argon atmosphere (red trace) or after vacuum drying (green trace). The inlet shows dried protein formulation on the sample holder after 20 hours of measurement at 253 K in vacuum.

Summarizing, diffractograms in Fig. S.4, Fig. S.5 and Fig S.6 show no signs of trehalose crystallization in the 160 mM trehalose solution nor in the protein formulation during cooling to 193 K or vacuum-drying. These results prove that trehalose crystallization is not the cause for aggregate formation.

***Determination of glass transition temperature T_g_’ of protein formulation:***

The glass transition temperature T_g_’ of the protein formulation was determined using DSC (Perkin Elmer, DSC8000). Hereby, 30 µl of sample was placed in a hermetically sealed aluminum capsule and cooled to – 90°C at rates of 5°C/min. After five minutes the sample was heated to 10°C at a rate of 5°C/min. The same freeze- and thaw-cycle was repeated three times. The T_g_’ of the protein formulation is seen in the thermogram of the heating curve (see Fig. S.7). The two glass transitions at – 30.5°C (T_g_’_1_) and – 34.4°C (T_g_’_2_) both originate from trehalose. The reason for the appearance of two transition events separated by ≈ 5°C may be two distinct types of freeze-concentration of trehalose.





**Fig. S.7:** Thermogram of protein solution containing sodium citrate buffer with 160 mM trehalose recorded at a heating rate of 5°C/min. Inlet shows magnified section of recorded thermogram.

***Optical cryomicroscopy images at* –** ***80°C:***

Morphology changes of ice crystals, size and orientation of ice crystals in the protein formulation were investigated by optical cryomicroscopy (OCM). A droplet containing the protein formulation was cooled to – 80°C at different rates, and images were recorded under standard transmitted light and crossed-polarized light (see Fig. S.8). It is difficult to see any morphology changes even with the highest magnification (50× ULWD objective, Olympus Corporation). The protein and the cryoprotectant trehalose covers, surrounds and coats the formed ice crystals. Within the frozen sample a thick intransparent network is formed (see Fig. S.8) which surrounds and covers the ice crystals. Different cooling rates did not change the outcome. The orientation of the ice crystals in the droplet, which can be visualized by using a crossed-polarized filter, was hardly visible.


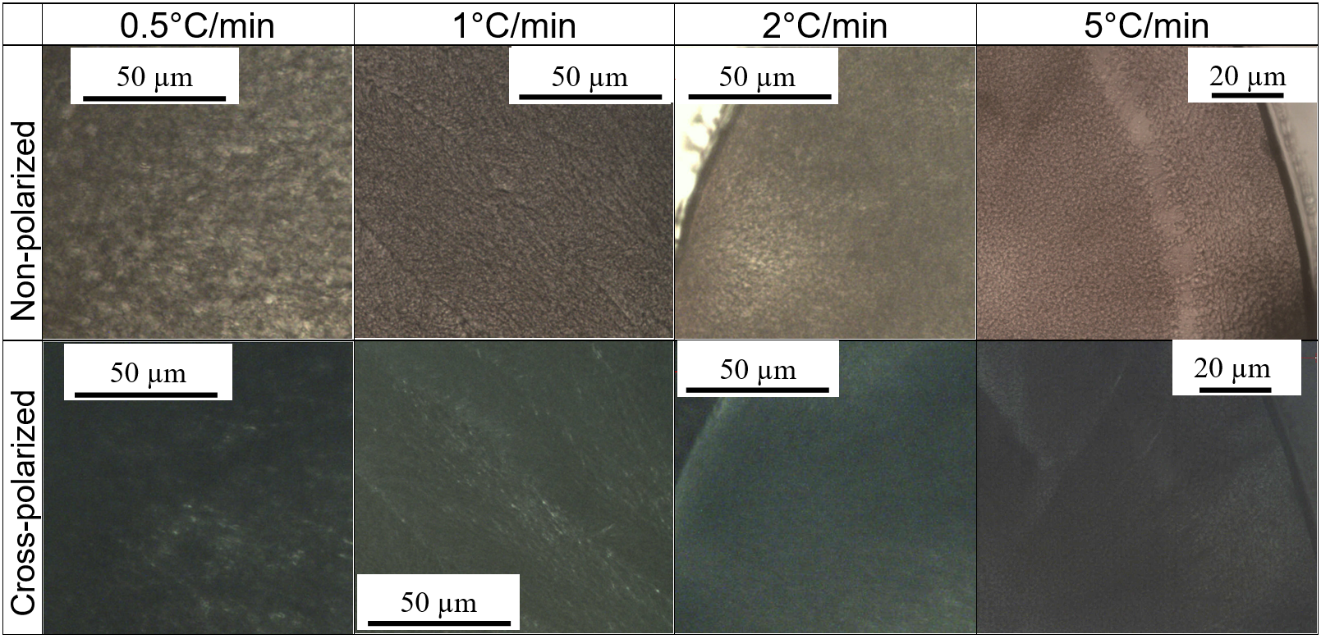


**Fig. S.8:** Optical cryomicroscopy images of protein solution containing sodium citrate buffer with 160 mM trehalose recorded at – 80°C after cooling at various rates (50× objective). The first row shows images recorded by using standard transmitted light without a filter and the second row shows images recorded by using a crossed-polarized filter.
